# Stem cell-derived extracellular vesicles: A novel and potential remedy for primary ovarian insufficiency

**DOI:** 10.3389/fcell.2023.1090997

**Published:** 2023-02-15

**Authors:** Zixiang Geng, Hailing Guo, Yifei Li, Ying Liu, Yongfang Zhao

**Affiliations:** ^1^ Shi’s Center of Orthopedics and Traumatology, Shuguang Hospital Affiliated to Shanghai University of Traditional Chinese Medicine, Shanghai, China; ^2^ Institute of Traumatology and Orthopedics, Shanghai Academy of Traditional Chinese Medicine, Shanghai, China; ^3^ Shanghai University of Traditional Chinese Medicine, Shanghai, China; ^4^ Department of Dermatology, Shanghai Songjiang District Central Hospital, Shanghai, China

**Keywords:** primary ovarian insufficiency, premature ovarian failure, stem cells, extracellular vesicles, exosomes

## Abstract

Primary ovarian insufficiency (POI) is an essential cause of young female fertility loss. At present, there are many treatments for primary ovarian insufficiency, but due to the complexity of the pathogenesis of primary ovarian insufficiency, the efficacy still could not be satisfactory. Stem cell transplantation is a feasible intervention protocol for primary ovarian insufficiency. However, its wide application in the clinic is limited by some defects such as tumorigenic and controversial ethical issues. Stem cell-derived extracellular vesicles (EVs) represent an important mode of intercellular communication attracting increasing interest. It is well documented that stem cell-derived extracellular vesicles for primary ovarian insufficiency with exciting therapeutic effects. Studies have found that stem cell-derived extracellular vesicles could improve ovarian reserve, increase the growth of follicles, reduce follicle atresia, and restore hormone levels of FSH and E2. Its mechanisms include inhibiting ovarian granulosa cells (GCs) apoptosis, reactive oxygen species, and inflammatory response and promoting granulosa cells proliferation and angiogenesis. Thus, stem cell-derived extracellular vesicles are a promising and potential method for primary ovarian insufficiency patients. However, stem cell-derived extracellular vesicles are still a long way from clinical translation. This review will provide an overview of the role and the mechanisms of stem cell-derived extracellular vesicles in primary ovarian insufficiency, and further elaborate on the current challenges. It may suggest new directions for future research.

## Introduction

Primary ovarian insufficiency (POI), also known as premature ovarian failure (POF) or premature menopause, is defined as cessation of menstruation before the expected age of menopause (Qin et al., 2015). In recent years, according to epidemiological surveys, POI is becoming a common disease in women’s reproductive systems, with an incidence rate of 1% (Szeliga et al., 2021). Studies have shown that 1 in 1,000 women between the ages of 15 and 29 and 1 in 100 women between the ages of 30 and 39 experience POI (Nippita and Baber, 2007; Panay and Fenton, 2008). There is a close relationship between ovarian granulosa cells (GCs) quality and the occurrence of POI (Zhu et al., 2021; Geng et al., 2022a) because senescence and cell cycle disorders in GCs result in a significant reduction of ovarian reserve (Fu et al., 2017; Liu et al., 2021). In clinics, hormone replacement therapy is the most commonly used management for POI patients but has a higher risk of various complications such as breast and ovarian cancer (Shelling, 2010; Kovanci and Schutt, 2015; Szeliga et al., 2021). Therefore, it is essential to find a safer and more effective way to treat POI.

Not surprisingly, stem cell-based therapies hold tremendous potential for treating POI in both preclinical and clinical trials (Ding et al., 2018; Yin et al., 2020; Fu et al., 2021; Mashayekhi et al., 2021; Wang et al., 2022a). Recent research suggests stem cells may provide therapeutic effects by paracrine means, specifically using extracellular vesicles (EVs) that include exosomes (Zhang et al., 2019a; Liu et al., 2021; Qu et al., 2022). The diameters of EVs range from approximately 30 nm–3,000 nm, and their biomolecular composition determines their functions, as well as their source and conditions (Riazifar et al., 2017). EVs are initially considered to be cellular debris or a way to remove toxic or unneeded by-products from the cell. Although EVs have ancient evolutionary origins and conserved mechanisms of generation, they play crucial physiological roles in cell-to-cell communication (Zhang et al., 2015; Xu et al., 2018). It is common for cells to secrete EVs, and these EVs can also be found in body fluids (Villarroya-Beltri et al., 2014; Xu et al., 2021). Substantial evidence has implicated that stem cell-derived EVs play an obvious role in the treatment of various diseases (Gnecchi et al., 2005; Clevers et al., 2014; Doeppner et al., 2015). It is worth noting that stem cell-derived EVs have shown promising results in treating POI. In this review, we summarize the applications of stem cells-derived EVs in POI and expound on the underlying cellular and molecular mechanisms. We also discuss the expectations for the future of stem cells-derived EVs.

## The difference between stem cells and stem cell-derived EVs

Stem cells have the potential for self-renewal and multidirectional differentiation, such as adipose stem cells (ADSCs), bone marrow mesenchymal stem cells (BMSCs), umbilical cord mesenchymal stem cells (UCMSCs) (Williams et al., 2008; Liao and Chen, 2014; Huang et al., 2021). Stem cells play vital roles in maintaining cellular homeostasis and restoring it upon tissue injury (Riazifar et al., 2017). Extensive research has shown that stem cells hold significant therapeutic potential in a variety of human diseases (Yamanaka, 2020). However, stem cells treatment carries an increased risk of conditions including organ failure and neurodegenerative disease (Poulos, 2018). Another potential safety risk for stem cell transplantation is increased immunogenicity (Nguyen et al., 2016). By far, the biggest concern is the tumorigenicity of stem cells due to their long-term culture, which may result in the accumulation of karyotypic abnormalities, copy number variation, and loss of heterozygosity (Lund et al., 2012). Hence, an increasing interest has been shifted toward stem cells-derived EVs. It is now clear from stem cell research that EVs are essential for cells to protect or regenerate injured cells, possibly through a paracrine effect mediated by the EVs (Han et al., 2021). In general, EVs, including exosomes and microvesicles (MVs), are membrane-enclosed vesicles containing proteins and nucleic acids (van Niel et al., 2018; Andaluz Aguilar et al., 2020). Exosomes are EVs with a size range of 40–160 nm (average 100 nm) in diameter with an endosomal origin (Kalluri and LeBleu, 2020). MVs, which vary from 50 to 1,000 nm in diameter, appear to have multiple points of origin, ranging from the selective outward pinching of the plasma membrane to membrane shedding and/or vesicles resulting from cell death (Stahl and Raposo, 2019). EVs comprise complex contents, such as nucleic acids including DNA, mRNAs, non-coding RNAs (ncRNAs), lipids, and various proteins (Mathivanan et al., 2010; Mashouri et al., 2019). Compared to stem cells, stem cell‐derived EVs possess multiple advantages including ethical access, abundant source, and low immunogenicity (Fang et al., 2020; Watanabe et al., 2021; Hade et al., 2022; Xia et al., 2022). Hence, stem cell‐derived EVs are considered to be a safer regenerative medicine approach for treating many otherwise untreatable diseases such as POI (Ding et al., 2020). But it should not be ignored that stem cells have the capacity for self-renewal, unlimited proliferation, and differentiation but not EVs.

## The pathogenesis of POI and the application of stem cell-derived EVs

The pathogenesis of POI has not been fully elucidated as it involves multiple factors including genetic, immunological, and environmental factors. A wide range of genetic defects is associated with POI, including X chromosome defects, which collectively account for 10%–25% of cases (Chen et al., 2018). More than 80 genes concern gonadal development, DNA replication/meiosis, DNA repair, and hormone synthesis (Franca and Mendonca, 2020). Furthermore, some POI patients suffer from autoimmune diseases, mainly thyroid autoimmune diseases (Dragojevic-Dikic et al., 2010). Common methods of anticancer treatment such as chemotherapy and radiotherapy could also cause female reproductive dysfunction (Meirow and Nugent, 2001). Cyclophosphamide and cisplatin are commonly used in clinical practice, and becoming the most common method of establishing an animal model of POI. In addition, other recognized causes of POI include metabolism disorders, infections, toxins, and environmental factors (Naleway et al., 2018; Moslehi et al., 2019; Rostami Dovom et al., 2019; El Bakly et al., 2020).

**FIGURE 1:**
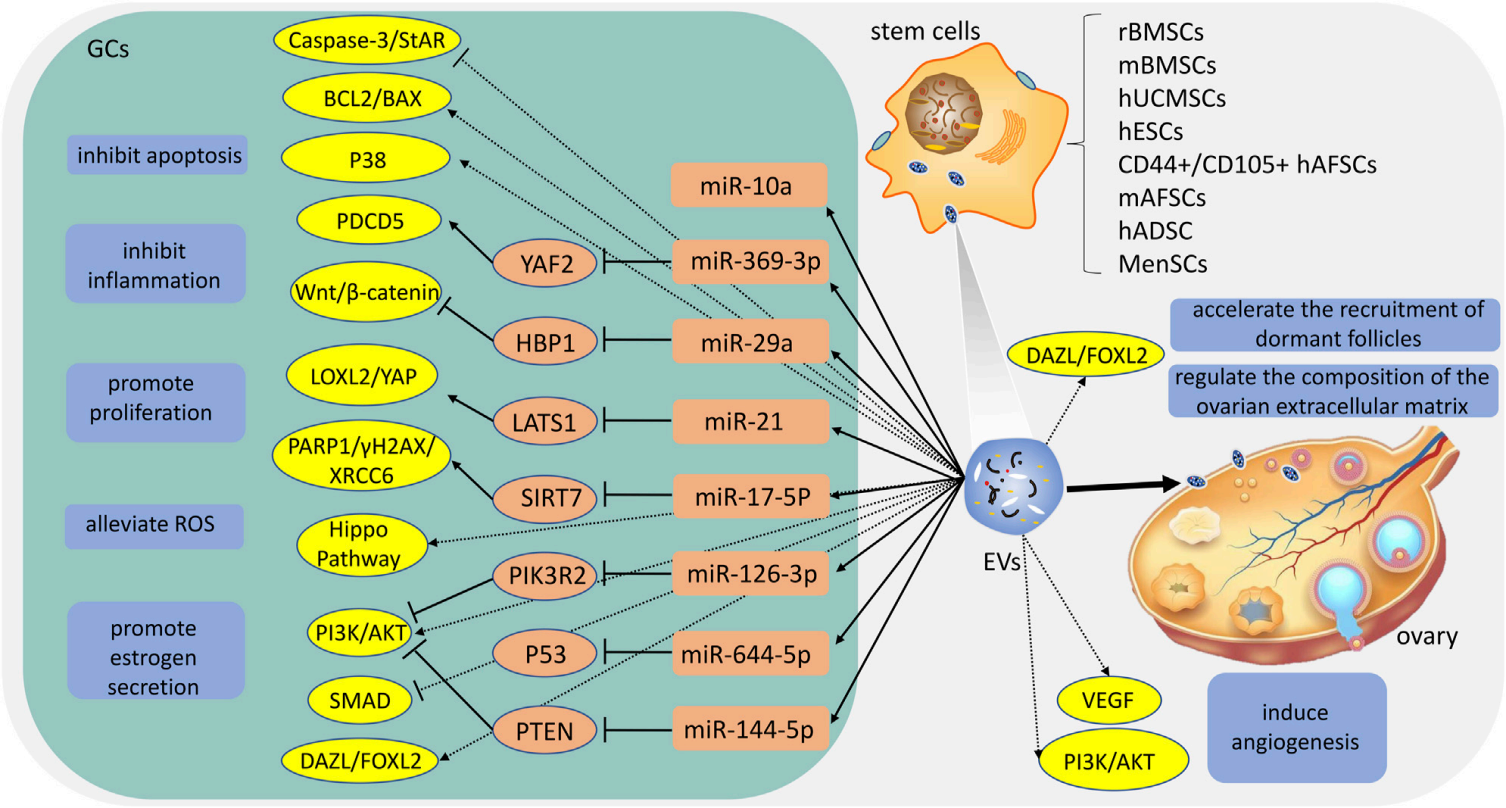
The potential mechanisms of different extracellular vesicles (EVs). rBMSCs: rat bone marrow mesenchymal stem cells; mBMSCs: mouse bone marrow mesenchymal stem cells; hUCMSCs: human umbilical cord mesenchymal stem cells; hESCs: human embryonic stem cells; hAFSCs: human amniotic fluid stem cells; mAFSCs: mouse amniotic fluid stem cells; hADSC: human adipose stem cells; MenSCs: menstrual blood-stem cells; ROS: reactive oxygen species; GCs: granulosa cells.

At present, there have been numerous researches on the treatment of stem cell‐derived EVs for POI and has received considerable clinical attention. Among these studies, sources of EVs include umbilical cord mesenchymal stem cells, bone marrow mesenchymal stem cells, embryonic stem cells, amniotic fluid stem cells, adipose stem cells, and menstrual blood stem cells (Ling et al., 2019; Tracy et al., 2019; Liu et al., 2020; Yang et al., 2020; Li et al., 2021a; Zhang et al., 2021) (Table 1). Studies have found that stem cell-derived EVs could improve ovarian reserve, increase the growth of follicles, reduce follicle atresia, and restore hormone levels of FSH and E2 (Fu et al., 2021). EVs contain a variety of lipids, nucleic acids, and proteins, and play an important role in cell-cell communication by transporting several molecules from donors to recipients (Mittelbrunn and Sanchez-Madrid, 2012). In particular, miRNA. miRNA is a distinct class of small (approximately 22 nucleotides), single-stranded, and non-coding RNAs, and play critical functions in the regulation of cellular gene expression by binding to complementary sequences in the target mRNAs, leading to either translational repression or target degradation of the specific mRNAs (Ha and Kim, 2014). Several studies indicate that miRNA carried by EVs plays a key role in the treatment of POI (Xiao et al., 2016; Ding et al., 2020; Geng et al., 2022b; Cai et al., 2022). In the following section, we explore the potential mechanisms and application value of different EVs (Figure 1).

**TABLE 1:** Preclinical studies of stem cells-derived EVs in POI.

EVs cellular origin	Model	Treatment	Functions	Pathways	Ref
rBMSCs	CTX-rat	150 μg EVs (100 μL PBS)/every other day for 2 weeks by intraperitoneal injection	inhibit GCs apoptosis	miR-144-5p *via* PTEN-PI3K/AKT	Yang et al. (2020)
mBMSCs	Cisplatin-mouse	125 μg EVs (100 μL PBS)/after model establishment 1st, 5th, and 10th day by tail vein injection	inhibit GCs apoptosis	miR-644-5p *via* P53	Sun et al. (2019)
hUCMSCs	CTX-mouse	1 × 10^6^ cells- EVs (200 μL PBS)/model establishment 8th, 9th day by intraperitoneal injection	Inhibit GCs apoptosis and inflammation	phosphorylation of AKT and P38	Deng et al. (2021)
		150 μg EVs/two times (Once every 7 days) by intraperitoneal injection	promote GCs proliferation	Hippo Pathway	Li et al. (2021a)
		10^11^, 5 × 10^11^, and 10^12^ cells- EVs particles/mL by intra-ovarian injection	promote GCs proliferation; alleviate ROS accumulation	miR-17-5P *via* SIRT7- PARP1/γH2AX/XRCC6	Ding et al. (2020)
	Cisplatin-rat	400 μg EVs (200 μL PBS)/after model establishment by tail vein injection	promote angiogenesis and attenuate GCs apoptosis	miR-126-3p *via* PIK3R2-AKT/mTOR	Qu et al. (2022)
	Cisplatin-rat GCs	30 μg/ml EVs	inhibit GCs apoptosis	Caspase-3; Bcl-2/Bax; StAR	Zhang et al. (2020)
	GCs (KGN and SVOG cells)	30 μg/ml EVs	promote GCs estrogen secretion	miR-21 *via* LATS1-LOXL2/YAP	Cai et al. (2022)
	Cisplatin-mouse	125 μg EVs (100 μL PBS)/after model establishment by tail vein injection	promote GCs proliferation; inhibit GCs apoptosis	miR-29a *via* HBP1-Wnt/β-catenin	Gao et al. (2022)
	Cisplatin-rat GCs	100 μg/ml EVs	inhibit GCs apoptosis	Caspase-3; Bcl-2/Bax; cleaved PARP	Sun et al. (2017)
	CTX/BUS-mouse	150 μg EVs (100 μL PBS)/once a week for 4 weeks by tail vein injection	induce angiogenesis	PI3K/AKT	Yang et al. (2019)
hESCs	CTX/BUS-mouse	1 × 10^8^ cells- EVs (200 μL)/once every 2 days for three times by tail vein injection	promote GCs proliferation; inhibit GCs apoptosis	PI3K/AKT	Liu et al. (2020)
CD44+/CD105+ hAFSCs	CTX-mouse	1 × 10^6^ cells- EVs/every 2 days for 4 weeks by tail vein injection	inhibit GCs apoptosis	miR-369-3p *via* YAF2- PDCD5/p53	Geng et al. (2022b)
mAFSCs	CTX/BUS-mouse	125 μg EVs by intra-ovarian injection	inhibit GCs apoptosis	miR-10a	Xiao et al. (2016)
hADSC	CTX-rat	intra-ovarian injection	inhibit GCs apoptosis; induce angiogenesis	VEGF; Bcl-2/Bax	Ling et al. (2019)
	CTX-mouse	1 × 10^6^ cells- EVs by intra-ovarian injection	inhibit GCs apoptosis; promote GCs proliferation;	SMAD pathway	Huang et al. (2018)
MenSCs	VCD-rat	25 μg EVs (50 μL) by intra-ovarian injection	promote GCs proliferation; regulate the composition of the ovarian extracellular matrix; accelerate the recruitment of dormant follicles	DAZL; FOXL2	Zhang et al. (2021)

EVs: extracellular vesicles; POI: primary ovarian insufficiency; rBMSCs: rat bone marrow mesenchymal stem cells; mBMSCs: mouse bone marrow mesenchymal stem cells; hUCMSCs: human umbilical cord mesenchymal stem cells; hESCs: human embryonic stem cells; hAFSCs: human amniotic fluid stem cells; mAFSCs: mouse amniotic fluid stem cells; hADSC: human adipose stem cells; MenSCs: menstrual blood-stem cells; ROS: reactive oxygen species; CTX: cyclophosphamide; VCD: 4-vinylcyclohexene diepoxide; BUS: busulfan; GCs: granulosa cells.

## Human umbilical cord mesenchymal stem cells-derived EVs (hUCMSCs-EVs)

It is the placenta that supplies foetal nutrition and connects the mother and the foetus during pregnancy (Tang et al., 2021). hUCMSCs-EVs can be isolated from various hUCMSCs compartments or the complete hUCMSCs. There are round or oval membranous vesicles, which can be aggregated and distributed, and their membrane structure is clearly defined (Vohra et al., 2020). hUCMSCs-EVs express the EVs-specific four-transmembrane protein markers CD9, CD63, CD81 and tumor susceptibility gene 101 protein (TSG101), heat shock protein70 (HSP70), and the multivesicular biosynthesis-related protein ALIX (Sun et al., 2017; Yang et al., 2019; Zhang et al., 2020; Deng et al., 2021; Cai et al., 2022; Gao et al., 2022; Qu et al., 2022). In 2017, hUCMSCs- EVs were first described for application to POI by Sun et al. (2017). Studies show that hUCMSCs-EVs ameliorate GCs stress and apoptosis *in vitro*, and the underlying mechanism may be related to the upregulation of BCL2 and the downregulation of BAX, cleaved caspase-3, and PARP (Sun et al., 2017; Deng et al., 2021). In addition, Sun et al. (2017) suggest that microRNA-24, microRNA-106a, microRNA-19b, and microRNA-25 may be closely related to apoptosis. After then, multiple studies show that hUCMSCs-EVs could restore serum FSH and estrogen levels, preserve the ovarian reserve and avoid antral follicle atresia. The mechanism involves the Hippo pathway and PI3K-AKT pathway (Yang et al., 2019; Li et al., 2021a). Li et al. (2021a) propose that when the key Hippo molecule (YAP) is blocked, hUCMSCs-EVs suppress the proliferation and function of ovarian cells by regulating the Hippo pathway. Moreover, complex ovarian vascular systems are critical for ovarian function and follicle development, which make the follicle and/or corpus luteum receive nutrients, oxygen, and hormone support, as well as synthesize and release steroids (Kamat et al., 1995; Robinson et al., 2009; Ezoe et al., 2014). Yang et al. (2019) consider that angiogenesis also plays a critical role in the application of hUCMSCs-derived EVs. Furthermore, the EVs-mediated transfer of miRNA is an important way by which stem cell function. Some miRNA including miR-126-3p, miR-21, miR-29a, and miR-17-5P carried by hUCMSCs-EVs could play a role at the post-transcriptional level (Ding et al., 2020; Cai et al., 2022; Gao et al., 2022; Qu et al., 2022). By binding to the 3′UTR of target genes, these miRNAs inhibit the expression of particular molecules to inhibit reactive oxygen species (ROS) production and apoptosis and promote cell survival, proliferation, and angiogenesis in GCs. It is worth mentioning that the protective effect of EVs on cisplatin-damaged GCs showed a dose-dependent effect. GCs are significantly more viable when 15 μg/mL of hUCMSC-EVs are added to their culture for 24 h; 25 g/mL is even more effective when administered for 48 h (Zhang et al., 2020).

## Bone marrow mesenchymal stem cells-derived EVs (BMSCs-EVs)

BMSCs is the first stem cell used to evaluate the efficacy in the treatment of POI (Fu et al., 2017). Studies have revealed that BMSCs or EVs infusion could decrease the expression of pro-inflammatory cytokines and oxidised biomolecules (He et al., 2018). BMSCs-EVs are cup-shaped or spherical in shape, with a clear model structure around them, and the diameters of EVs distribution ranged between 30 and 2,000 nm (Li et al., 2021b; Yu et al., 2021). BMSCs-EVs express the EVs-specific four-transmembrane protein markers CD9, CD63, CD81 and TSG101, HSP70, and the multivesicular biosynthesis-related protein ALIX (Tang et al., 2021). Moreover, BMSCs-EVs are negative for CD14, CD34, and CD45 (Li et al., 2019; Tang et al., 2021). BMSCs-EVs appear to have a significant anti‐apoptotic effect *in vitro* and *in vivo* (Su et al., 2021; Wen et al., 2021; Xiong et al., 2021; Wang et al., 2022b). Sun et al. (2019) and Yang et al. (2020) also suggest that BMSCs-EVs inhibit GCs apoptosis by carrying miR-144-5p and miR-644-5p to inhibit PTEN and p53.

## Embryonic stem cells-derived EVs (ESCs-EVs)

Embryonic stem cells, derived from the blastocyst stage embryos, are distinguished by their ability to self-renew and differentiate into all cell types (Ohtsuka and Dalton, 2008; Martello and Smith, 2014). Thus, ESCs-EVs are extensively studied. Many studies have shown that ESCs-EVs can suppress senescence, facilitate cell proliferation, and inhibit cell apoptosis and oxidation (Khan et al., 2015; Bae et al., 2019; Zhang et al., 2019b; Tavakoli Dargani and Singla, 2019; Abbaszadeh et al., 2020). But ESCs-EVs are poorly studied in POI. Only one study suggests that ESCs-EVs could improve ovarian function by regulating the PI3K/AKT signaling pathway (Liu et al., 2020). Due to ESCs exhibiting strong self-renewal capability and pluripotency, ESCs-EVs are worth further exploration in POI.

## Amniotic fluid stem cell-derived EVs (AFSCs-EVs)

Amniotic fluid is a rich source of stem cells that can be easily obtained through amniocentesis during standard prenatal care procedures (Di Trapani et al., 2015). The procedure to obtain AFSCs is non-invasive, safe, and without social controversy (Soncini et al., 2007; Parolini et al., 2008; Diaz-Prado et al., 2010). Hence, amniotic fluid stem cells seem to be the optimal source of EVs. Xiao et al. (2016) reveal that mouse AFSC-EVs contained two microRNAs (miRNAs), miR-146a and miR-10a, which inhibited apoptosis in damaged GCs and prevented ovarian follicles from atresia in mice following cyclophosphamide (CTX). Liu et al. (2012) find that CD44+/CD105+ human AFCs possess the characteristics of mesenchymal stem cells (Zou et al., 2011) and it can survive and proliferate over the long term in the ovarian tissues of a mouse model of chemotherapy-induced POI . Subsequently, AFSCs-EVs are isolated and used in POI. Geng et al. (2022b) indicate that CD44+/CD105+ human AFSC-EVs carrying miR-369-3p could specifically downregulate the expression of YAF2, inhibit the stability of PDCD5/p53, and reduce the apoptosis of OGCs, thereby exerting therapeutic effects on POI. In addition, compared with BMSCs, AFSCs secreted higher levels of EVs (Tracy et al., 2019). Therefore, AFSCs-EVs may be more valuable than other EVs.

## Human adipose stem cells-derived EVs (hADSC-EVs)

It is widely accepted that adult stem cells can be found in abundance, readily accessible, and replenishable in adipose tissue. ADSCs are obtained from the subcutaneous adipose tissue removed during liposuction surgeries or abdominoplasties (Fu et al., 2021). Nowadays, ADSCs are widely used to treat various ailments of the skin. However, few studies have been reported on POI. Huang et al. (2018) reveal that the hADSC-EVs recover the ovarian function of POI by downregulating SMAD2, SMAD3, and SMAD5 expression. HADSC-conditioned media containing various cytokines and microvesicles secreted by HADSCs is concentrated and injected into the bilateral ovaries of POI rats in one study. The results show that hADSC-conditioned media injection partially reduces ovarian injury and improved ovarian function in rats with POI (Ling et al., 2019). We have reasons to believe that hADSC-EVs play an indispensable role. Certainly, more evidence is needed to demonstrate these linkages.

## Menstrual blood stem cells-derived EVs (MenSCs-EVs)

MenSC-derived small EVs were first reported in 2016 (Lopez-Verrilli et al., 2016), and the authors revealed that MenSC-derived small EVs promote axonal regeneration after nerve injury in the central and peripheral nervous systems. MenSCs-EVs are still in the early stages of study, unlike some common MSC sources such as bone marrow, umbilical cord, and adipose tissue (Chen et al., 2021). Until now the only evidence suggests that GCs were proliferated in primordial and primary follicles by MenSCs-EVs, and apoptosis was inhibited. MenSCs-EVs also increased the expression of early follicle markers, for example, DAZL and FOXL2 (Zhang et al., 2021). *In vivo*, transplantation of MenSCs-EVs in the rat model of POI promoted follicle development and restored estrous cyclicity and serum sex hormone levels. In addition, by transplanting MenSCs-EVs, the extracellular matrix of the ovary was regulated and dormant follicles were recruited sooner. As a result, MenSCs-EVs significantly promoted follicle development *in vitro* and *in vivo* and restored fertility in POI rats.

## Future challenges of stem cell-derived EVs

In the last few years, stem cell-derived EVs have emerged as a new therapeutic strategy for many diseases. Although multiple preclinical studies have shown that stem cell-derived EVs have positive effects in treating POI, it is far from being sufficient. Therefore, in order to benefit POI patients as quickly as possible, further pre‐clinical and clinical studies are warranted. But the optimal source of EVs should be found before this time. Regrettably, there are no studies to compare the therapeutic effects of different EVs for POI. Moreover, due to the ultra-short duration of the development of EVs, their safety and consistent regulatory issue are not conclusive (Hu et al., 2022).

To isolate EVs from various cell types, tremendous effort has been devoted in the past. Currently, there is numerous separative technique of EVs including precipitation, membrane affinity, size-exclusion chromatography, iodixanol gradient, and phosphatidylserine affinity (Lee et al., 2021). But no method is perfect. Furthermore, many studies have reported the Effect of storage temperature and frozen/thawed cycles on EVs size and biological activity, and 80°C was chosen as the optimum temperature to ensure both treatment outcomes and transport capacity (Kusuma et al., 2018; Le Saux et al., 2020). It is also one of the main challenges for EVs to mass culture. The strategies for mass-production of EVs include modulating the components or secretary machinery proteins of EVs, increasing the intracellular Ca ions, adjusting biochemical cues such as extracellular DNA, liposomes, and proton concentration, and applying physicomechanical cues such as forces and other stimuli (e.g., electricity, thermal, photodynamic, and radiative stress) (Lee et al., 2021). It is worth mentioning that the development of nanotechnology and biomaterials provide a viable means with which to tackle the previously mentioned problems (Lee et al., 2021). However, related studies were not available in POI.

In conclusion, although stem cell-derived EVs hold great prospects in treating POI, the following questions also need to be addressed: 1) the optimal source of EVs; 2) the safety of stem cell-derived EVs; 3) the mass cultivation and preservation of stem cell-derived EVs; 4) the clinical evaluation of stem cell-derived EVs; 5) the mechanism of stem cell-derived EVs.

## Conclusion

It is evident that stem cell-derived EVs have the potential in treating POI. However, more research was needed to investigate the mechanism of stem cell-derived EVs in POI and more efficient means for obtaining and preserving EVs to make benefit patients safer and faster.
